# Tafenoquine: A Breakthrough Option for Babesiosis Treatment

**DOI:** 10.3390/tropicalmed11050141

**Published:** 2026-05-19

**Authors:** Dongxue Ma, Mo Zhou, Shinuo Cao, Eloiza May Galon-Bedonia, Zhiqiang Xu, Chenghui Li, Xu Gao, Shujiang Xue, Shengwei Ji

**Affiliations:** 1 Department of Veterinary Medicine, Agriculture College, Yanbian University, Yanji 133000, China; madongxueyjsz@163.com (D.M.); 0000008040@ybu.edu.cn (Z.X.); lichenghuivip0056@163.com (C.L.); gaoxu@ybu.edu.cn (X.G.); sjxue@ybu.edu.cn (S.X.); 2 Swine Infectious Diseases Division, Jiangsu Key Laboratory for High-Tech Research and Development of Veterinary Biopharmaceuticals, Engineering Technology Research Center for Modern Animal Science and Novel Veterinary Pharmaceutic Development, Jiangsu Agri-Animal Husbandry Vocational College, Taizhou 225306, China; zhoumo_wk@hotmail.com (M.Z.); shinuo_cao@163.com (S.C.); 3 Department of Basic Veterinary Sciences, College of Veterinary Medicine and Biomedical Sciences, Cavite State University, Cavite 4122, Philippines; eloiza.galon@cvsu.edu.ph

**Keywords:** tick-borne diseases, babesiacides, drug resistance, novel drug, tafenoquine

## Abstract

Babesiosis is a zoonosis caused by protozoan parasites of the genus *Babesia*. It has a worldwide distribution and affects many kinds of mammals, principally domestic animals and humans. Because there are no safe and effective vaccines available, the treatment and control for babesiosis continues to involve the use of chemotherapeutics. For years, only a few drugs have been used for clinical treatment, namely atovaquone plus azithromycin or clindamycin plus quinine for human, and imidocarb dipropionate and diminazene aceturate for domestic animals. Although screening and developing alternative drugs are continuously pursued, only a few drugs have been prospected to have clinical applications. Of these, tafenoquine has shown wide and potent antibabesial activity, offering a new option to control babesiosis. This article aims to present the current clinical therapeutic strategies for babesiosis and their limitations, as well as the prospect of tafenoquine as a promising drug to treat babesiosis.

## 1. Introduction

Babesiosis is a global emerging tick-borne disease caused by infection with intra-erythrocytic parasites of the genus *Babesia*. It exerts substantial economic, veterinary and medical impact worldwide [1,2]. From the first case report in cattle in 1888, over 100 known *Babesia* species, mostly host-specific, have been identified across a wide geographical range. These species could infect many types of mammalian hosts, including humans, pets, domestic animals, wild animals [1].

The first case of human babesiosis was described in 1957 in the former Yugoslavia (now Croatia), involving a splenectomized patient who died after an acute illness [3]. Since then, several species have been identified as causative agents of human babesiosis, including *Babesia microti*, *B. duncani*, *B. divergens*, *B. divergens*-like, *B. crassa*-like, *B. venatorum*, *Babesia* sp. KO-1, and *Babesia* sp. XXB/HangZhou (the latter exclusively occurs in Asia); these species have distinct geographic distributions [4,5].

Over the past few decades, human babesiosis cases caused by *B. microti* have increased exponentially, particularly in the northeastern and northern midwestern regions of the U.S. [5]. Fu et al. [6] presented a review on the global distribution of the 26,848 recorded human babesiosis cases. More than 95% of cases were caused by *B. microti*, followed by *B. duncani* [6]. In animals, several species can infect domestic animal, of which bovine-infective species (*B. bovis*, *B. bigemina*, and *B. divergens*) inflict severe economic impact on livestock industries. Notably, *B. divergens* warrants particular attention as a major zoonotic species. A primary cause of bovine babesiosis, it is also capable of causing life-threatening infections in humans, especially in splenectomized individuals [7]. Globally, more than 500 million cattle are exposed to babesiosis infection, not only threatening animal health but also posing a substantial threat to human livelihoods [8,9].

The multi-host and complex nature of the life cycle of *Babesia* allow for targeting various hosts and distinct parasite stages. The current strategies for identifying effective treatment includes both novel compound discovery and the strategic repurposing of existing drugs, particularly other parasiticides. However, the emergence of drug-resistant parasites represents a significant challenge in the control of babesiosis. This paper sought to provide insights into current clinical therapeutic strategies for human and animal babesiosis and their limitations, while highlighting tafenoquine as a breakthrough candidate to address challenges of toxicity and resistance.

## 2. Parasite Life Cycle and Clinical Manifestations

**Figure 1:**
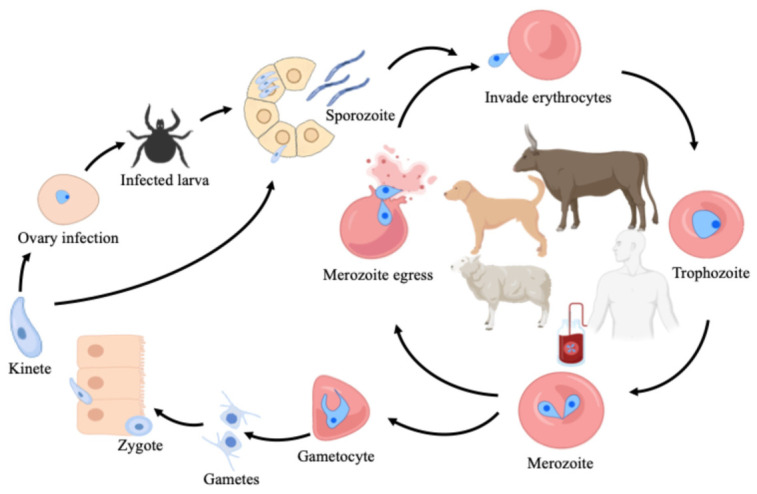
The general life cycle of *Babesia* species. The cycle begins when an infected tick inoculates sporozoites into a host during feeding. These parasites invade red blood cells to undergo merogony, producing merozoites that either continue infecting new cells or differentiate into gametocytes. When another tick feeds, it ingests these gametocytes, differentiating into gametocytes which then fuse into zygotes within the tick’s midgut. These zygotes eventually develop into new sporozoites in the salivary glands, completing the cycle.

The general life cycle of *Babesia* spp. includes three phases: gamogony, sporogony, and merogony [1]. In general, ticks become infected after ingesting a blood meal containing gametocytes from a mammalian host. Within the lumen of the tick’s midgut, the gametocytes differentiate into gametes, which fuse to form zygotes. These zygotes invade the midgut epithelial cells to develop into motile kinetes, undergoing initial multiplication, and then are released into the hemolymph. These then migrate to various tissues, such as the ovaries (transovarial transmission) and salivary glands. Upon reaching the salivary glands, these parasites undergo sporogony to generate infectious sporozoites. Following a subsequent tick bite, these sporozoites invade red blood cells (RBCs) and develop into trophozoites. Subsequently, merozoites that formed from trophozoites egress from the ruptured RBCs to invade new cells [10]. This process repeats, while some of the merozoites differentiate into gametocytes, which infect feeding ticks [11]. Notably, human to human transmission can occur via blood transfusion or from an infected pregnant woman to the fetus [12] (Figure 1).

The clinical manifestations range from asymptomatic infection to severe, fatal disease. Patients often exhibit malaria-like symptoms, including fever, fatigue, chill, and anorexia [1,13]. The severity of *Babesia* infection is strongly associated with host immunity. Although symptoms may resolve spontaneously in immunocompetent patients without any treatment, severe babesiosis typically occurs in immunocompromised patients, i.e., patients with HIV infection, advanced age, asplenia, and immunosuppressive therapy [14,15]. In animals, *Babesia* infection is exhibited as high fever, anorexia, weakness, anemia, jaundice, and hemoglobinuria [16].

## 3. Conventional Drugs for Human Babesiosis

Most asymptomatic patients do not require treatment. Immunocompetent patients are treated orally with atovaquone plus azithromycin for at least 7–10 days, while for highly immunocompromised patients, this may be extended to at least 6 weeks [17]. This combination is recommended for all *B. microti*-infected patients as it presents fewer side effects than clindamycin plus quinine, which is often reserved when parasitemia and symptoms persist despite treatment with atovaquone plus azithromycin [17]. However, in severe cases, a prolonged treatment period selects for *cytochrome b* (*Cytb*) and *ribosomal protein subunit L4* (*RPL4*) mutations, which confers drug resistance to the parasites [18,19]. Consequently, if initial antibabesial therapy fails, patients may experience a prolonged relapsing course of disease. While clindamycin plus quinine remains the standard of care for severely ill patients, this drug combination shows serious side effects to humans, such as headache, hearing loss, dizziness, and tinnitus [20]. Furthermore, monotherapy with clindamycin or quinine seems to have poor efficacy against *Babesia* [21]. A critical limitation of these conventional regimens is their frequent failure to achieve complete parasitological clearance in immunocompromised individuals, leading to persistent low-grade parasitemia and subsequent clinical relapses [22]. This inability to eliminate the reservoir of infection not only necessitates prolonged treatment but also facilitates the selection of drug-resistant strains [19].

## 4. Conventional Drugs for Animal Babesiosis

**Figure 2:**
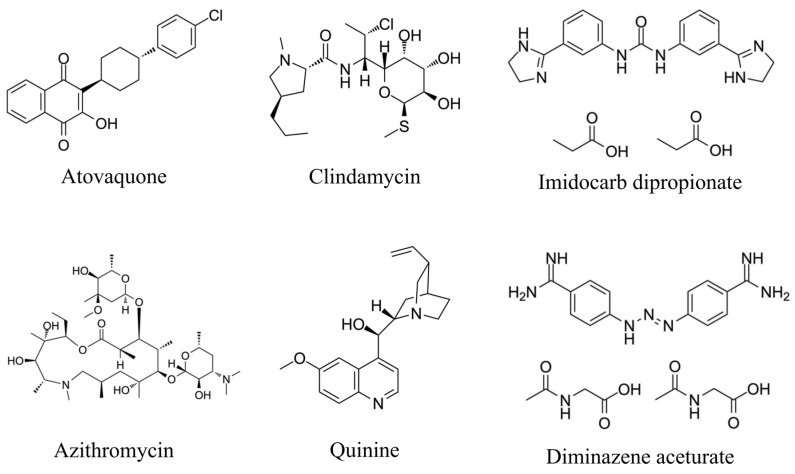
Chemical structures of commonly used antibabesial compounds.

Imidocarb dipropionate and diminazene aceturate are the most commonly used commercial drug for animal babesiosis (Figure 2). These compounds are often used in adult or mature animal populations, who are more susceptible to acute or clinical babesiosis, as majority of infected young ruminants remain subclinical and develop natural immunity upon infection [23].

Imidocarb dipropionate, which was developed in the 1970s, possesses broad inhibitory activity against several *Babesia* spp., including *B. bovis*, *B. bigemina*, *B. divergens*, *B. caballi*, at a dose of 1–3 mg/kg [14]. A higher dose of 6.6 mg/kg has been approved by the U.S. FDA for the treatment of *B. canis* infection [24]. It has been widely used in the treatment and prophylaxis of animal babesiosis and anaplasmosis in the past few decades. However, imidocarb residues can persist in the edible tissues of ruminants for long periods, raising concerns regarding food safety [25,26,27]. Diminazene aceturate has been marketed for over six decades and is a widely used compound that controls babesiosis and trypanosomiasis [28,29]. It has potent inhibition against *B. bigemina*, *B. bovis*, *B. caballi*, *B. gibsoni*, and *B. canis* at a dose of 3–5 mg/kg [14,24,30,31]. Despite its long-term use in clinical cases, the exact mechanism of diminazene remains largely unknown. A series of studies indicated that diminazene resistance in *B. gibsoni* is not directly related to the mitochondrial genes or the activation of glycolysis [32,33,34]. Long-term use has led to resistance in several species of *Trypanosoma* [35] and *Babesia* [32,36]. Furthermore, diminazene causes adverse effects, including severe and unpredictable neurological signs even at therapeutic doses [24].

## 5. Introduction of Tafenoquine

Tafenoquine, also known as WR238605 or SB-252263, is an 8-aminoquinoline anti-malarial drug originally developed by the Walter Reed Army Institute of Research and GlaxoSmithKline in 1978 [37]. Tafenoquine was approved by the U.S. Food and Drug Administration (FDA) in 2018 for the radical cure of *Plasmodium vivax* infection and malaria chemoprophylaxis [38]. Currently, tafenoquine is marketed under the name of Krintafel^TM^ (150 mg tablets) for *P. vivax* treatment in patients over 16, and Arakoda^TM^ (100 mg tablets) for malaria prophylaxis in adults [39]. While primaquine has been the first-line drug for elimination of *P. vivax* hypnozoites since the 1950s, its short half-life, required 14-day administration period, and increasing cases of primaquine tolerance prompted the search for alternative compounds [40]. As a synthetic analog of primaquine, tafenoquine demonstrates greater activity against blood- and liver-stages of the parasites [41]. Its longer half-life allows for simplified dosing regimens leading to fewer side effects, suggesting that tafenoquine is a promising drug for malaria treatment and prevention [41].

## 6. Chemistry of Tafenoquine

**Figure 3:**
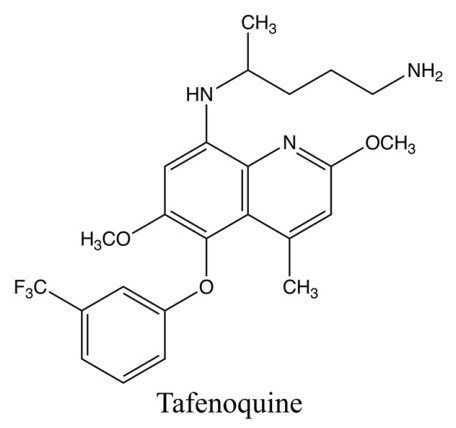
Chemical structure of tafenoquine.

The chemical name of tafenoquine is 8-([4-amino-1-methylbutyl]amino)-2,6-dimethoxy-4-methyl-5-([3-trifluoromethyl]-phenoxy) quinoline succinate (Figure 3). Its chemical formula is C_24_H_28_N_3_O_3_·C_4_H_6_O_4_, with molecular weights of 463 for the base and 581 for the succinate salt [38]. Tafenoquine has an additional methoxy group at the 2-position, a methyl group at the 4-position, and a 3-trifluoromethylphenoxy substitution at the 5-position of the quinoline ring. In addition, the 3-trifluoromethylphenoxy substitution at the 5-position is related to the longer half-life and decreased potency for the formation of methemoglobin compared with primaquine [42].

## 7. Pharmacodynamics and Pharmacokinetics

Tafenoquine is active against all human life cycle stages of malaria parasites [41,43,44]. Although the exact mode of action is not fully understood [45], it is known to inhibit hematin polymerization, like chloroquine [46]. In addition, the side chain of 4-amino-1-methylbutyl is associated with the generation of super oxides, which may target exoerythrocytic parasites [47]. Part of its efficacy is due to its triggering of parasite apoptosis; tafenoquine inhibits cytochrome c reductase (respiratory complex III), interfering with the function of mitochondria. This causes decreased oxygen consumption and depolarization of mitochondrial membrane potential, resulting in reactive oxygen species (ROS) generation, elevation of intracellular Ca^2+^ levels, and concomitant nuclear DNA fragmentation [48,49,50]. This process likely applies in *Babesia* spp., where the accumulation of ROS results in vacuole-like aberrant appearance and death of parasites [51,52]. Tafenoquine is delivered orally and absorbed slowly; absorption increases when taken with food [41]. The half-life of tafenoquine in rodents, dogs and monkeys were 60 h, 170 h, and 52 h, respectively [53]. In humans, following a single 200 mg oral administration, tafenoquine reaches a peak plasma concentration of 113–181 ng/mL within 12 to 24 h. The drug exhibits a prolonged average terminal half-life of approximately 14 to 15 days [54]. Owing to its high lipophilicity, the drug exhibits an extensive apparent volume of distribution and is predominantly bound to plasma proteins, ensuring prolonged tissue residence. Its metabolism is partially mediated by *cytochrome* P450 2D6 (CYP2D6), which is essential for its hypnozoiticidal activity, while systemic elimination occurs slowly via the biliary–fecal route with minimal renal involvement [42,54].

## 8. Safety and Tolerability

Common adverse effects of tafenoquine include weakness, vertigo, dizziness, headache, and decreased hemoglobin [42,55,56,57,58]. The potential adverse effects of tafenoquine were evaluated in 369 participants with a weekly prophylactic dose ranging from 25 mg to 200 mg. Gastrointestinal abnormalities were the predominant adverse events, occurring in 13% to 18% of subjects. Importantly, the study confirmed the absence of a dosage-dependent relationship with either physical complaints or abnormal laboratory parameters, such as alanine aminotransferase, hemoglobin, white blood cell counts, platelet counts, and bilirubin levels. In a 6-month safety trial involving 492 participants receiving 200 mg of TQ weekly, 13% (66 participants) reported at least one adverse event, with nausea vertigo, diarrhea, and abdominal pain being the most common [59]. The most important concern for 8-aminoquinolines-based therapy is glucose-6-phosphate dehydrogenase (G6PD) deficiency [60,61,62], which is the most commonly inherited X-linked recessive disorder. As a critical enzyme for the generation of NADPH via the pentose phosphate pathway (especially in mature erythrocytes), G6PD deficiency precludes reduction of the effects of oxidative stress resulting in hemolytic anemia in humans [45,63]. Therefore, G6PD deficiency testing is mandatory prior to tafenoquine prescription.

## 9. Efficacy of Tafenoquine on Babesiosis

The efficacy of tafenoquine in *Babesia* spp. was first evaluated against *B. microti* in a hamster model, where a dose of 52 mg/kg twice a day for 4 days eradicated the parasite [64]. Tafenoquine demonstrated potent activity against *B. microti* in severe combined immunodeficiency (SCID) mice with a single dose of 15.9 mg/kg [52]. Furthermore, it also showed excellent efficacy against experimental *B. gibsoni* infection in immunocompromised dogs and naturally infected, clinical cases [51,65]. Although relapses were observed in both studies, tafenoquine was generally well tolerated and may achieve radical cure of babesiosis through an optimized dosing regimen.

Recently, the first human case of *B. microti* treated with tafenoquine was reported in an immunocompromised patient with a history of rituximab treatment who was resistant to both atovaquone and azithromycin. Treatment with tafenoquine with a course of 200 mg for 3 consecutive days followed by 200 mg once per week resulted in negative blood smears and PCR within 6 weeks (Table 1). Although this efficacy may be partially attributed to immune recovery (as evidenced by seropositivity for *B. microti*), the tolerability and the once-per-week single-drug dosing regimen are notable in the treatment of patients with babesiosis [66].

Rogers et al. [67] described a relapsing *B. microti* infection case in an immunocompromised patient with molecular evidence of antimicrobial resistance. A loading dose of 600 mg over 3 days (300 mg for day 1 and 150 mg for days 2 and 3), followed by a maintenance dose of 300 mg weekly was well tolerated and resulted in a clinical cure after 60 days of therapy. However, treatment is not always successful. One case failed to cure an immunocompromised patient after a course of 42-day tafenoquine treatment (200 mg for 3 consecutive days followed by 200 mg once per week). The treatment was discontinued due to unexpected adverse reaction and relapse evidenced by a positive PCR result by 21 days following discontinuation of treatment [68].

The potential for tafenoquine to achieve a radical cure was initially demonstrated in a hamster model, where specific dosing eradicated *B. microti* [64]. Clinical data on tafenoquine for human babesiosis are limited. However, clinical outcomes in humans have been heterogeneous. Some immunocompromised patients with multi-drug-resistant babesiosis achieve full parasite clearance, whereas others relapse after stopping treatment. Krause et al. [69] reported five cases of relapsing human babesiosis treated with tafenoquine (including one case described above). In four out of five, clearance was achieved after tafenoquine monotherapy or in combination with other antimicrobials; one highly immunocompromised patient experienced relapse after tafenoquine monotherapy. Hence, tafenoquine-based therapies may be warranted for immunocompromised patients with relapsing babesiosis [69,70]. Determining whether tafenoquine can consistently eliminate the parasite across different host immune statuses is essential for its potential role in public health strategies aimed at reducing disease prevalence.

## 10. Conclusions and Future Direction

Despite traditional recommendations remaining stagnant for years, rising drug resistance and severe adverse effects have made the search for novel therapies vital to reduce babesiosis-associated mortality. This paper provides an overview of tafenoquine, covering its chemistry structure, pharmacodynamics and pharmacokinetics, safety and tolerability, and the presumptive mode of action on *Babesia* spp. As a promising antibabesial compound, tafenoquine shows several advantages: (1) a single dose of tafenoquine demonstrates potent inhibition against *Babesia* spp., which may reduce the risk of drug-resistance development associated with frequent dosing; (2) tafenoquine-based therapies have the potential to radically cure babesiosis in immunocompromised hosts; and (3) tafenoquine could be a promising option for the prevention of babesiosis in endemic areas. Future research should prioritize evaluating the capacity of tafenoquine to achieve a radical cure rather than suppressive therapy, while expanding efficacy trials to include zoonotic species such as *B. divergens*.

Although tafenoquine was approved by the FDA for use in malaria treatment, it has yet to be approved for human babesiosis. While a phase II clinical trial is currently recruiting candidates, its clinical development should be accelerated, alongside explorations into TAF-based combination therapies to combat the issue of development of resistance. Such efforts hold the potential to revolutionize the treatment of babesiosis in the near future.

## Figures and Tables

**Table 1:** Summary of clinical cases using tafenoquine for relapsing babesiosis.

Case Description	TAF Regimen	Result	Ref
36-year-old male with granulomatosis with polyangiitis and prior rituximab therapy.	200 mg daily for 3 days, then 200 mg weekly for 6 weeks.	Cured	[66]
80-year-old male with monoclonal B-cell lymphocytosis treated with rituximab and bendamustine.	300 mg day 1, 150 mg days 2–3, then 300 mg weekly.	Cured	[67]
74-year-old female with diffuse large B-cell lymphoma (prior rituximab) and cold autoimmune hemolytic anemia.	200 mg daily for 3 days, then 200 mg weekly for 46 days (discontinued early due to neutropenia).	Relapsed	[68]
56-year-old male with splenectomy and chemotherapy for abdominal sarcoma.	300 mg day 1, 150 mg days 2–3, then 300 mg weekly.	Cured	[69]
52-year-old male with follicular lymphoma treated with rituximab-based regimen.	300 mg day 1, 150 mg days 2–3, then 300 mg weekly.	Cured	[69]
70-year-old female with rheumatoid arthritis treated with rituximab and methotrexate, and prior splenectomy.	200 mg daily for 3 days, then 200 mg weekly.	Cured	[69]
58-year-old male with untreated chronic lymphocytic leukemia.	300 mg day 1, 150 mg days 2–3, then 300 mg weekly.	Relapsed	[69]

## Data Availability

The original contributions presented in this study are included in the article. Further inquiries can be directed to the corresponding author.
